# The Role of Echocardiography in the Diagnosis of Left Ventricular Noncompaction: Usefulness in a Resource‐Constrained Setting

**DOI:** 10.1002/ccr3.9563

**Published:** 2024-12-12

**Authors:** Aba A. Folson, Philip Eghan, Daniel Amenu

**Affiliations:** ^1^ School of Medicine University of Health and Allied Sciences Ho Ghana; ^2^ University of Ghana Medical Centre Accra Ghana; ^3^ Ho Teaching Hospital Ho Ghana

**Keywords:** echocardiography, Ghanaian, left ventricular, noncompaction

## Abstract

Left ventricular non compaction is a genetic cardiomyopathy with a high occurence in individuals of African ancestry and may present in adulthood with diagnostic challenges when there is advanced heart failure. Echocardiography and Magnetic Resonance Imaging have mostly been used in making a diagnosis. However, there is a lack of these diagnostic tools required to aid in early diagnosis in Low and Middle Income Countries. The potential usefulness of echocardiography in this population is the focus of this case presentation.

**Summary**

- Genetic cardiomyopathies such as left ventricular noncompaction (LVNC) are difficult to diagnose in low‐resource settings due to a lack of advanced diagnostic equipment.
- Enhanced awareness of the typical echocardiographic features of LVNC and diligent application will improve the likelihood of early diagnosis of index cases and screening of first‐degree relatives by cardiologists despite these limitations.

## 1 Introduction

Left ventricular noncompaction (LVNC), also referred to as spongiform cardiomyopathy, is a congenital cardiomyopathy with an estimated prevalence between 0.01% and 0.14%, but a high occurrence in individuals with African ancestry [1, 2]. The condition is characterized by noncompacted left ventricular myocardium with trabeculations and deep intertrabecular recesses following cessation of myocardial compaction process during embryonical development [3, 4]. In a retrospective study carried out in Senegal, the mean age of patients with LVNC was 47 ± 18.4 years with the commonest echocardiographic abnormality being left ventricular hypertrophy (46%), while 77% of them were found to be in heart failure [4].

Echocardiography is the first‐choice diagnostic modality, complemented by other advanced imaging modalities such as cardiac magnetic resonance (CMR) [5]. Multiple criteria have been developed in the diagnostic approach using echocardiography. In a review by Peters and Essop [6], most case studies in Africa used a combination of the Jenni and Stolberger criteria in diagnosing LVNC. These two criteria proposed the understated echocardiographic findings as consistent with LVNC.

1. Prominent left ventricular trabeculations, predominantly in the apical and midventricular areas of both the inferior and lateral walls.
2. The two‐layered appearance of the myocardium with a thin, compacted outer band and a thicker, noncompacted inner layer. The end‐systolic ratio between noncompacted and compacted myocardium is expected to be > 2.
3. Multiple deep intertrabecular recesses communicate with the ventricular cavity, as visualized by color Doppler imaging and the absence of additional coexisting cardiac abnormalities.
4. Lastly, the end‐diastolic ratio between noncompacted and compacted myocardium should be > 2 in the apical four‐chamber view [7].

We present the case of a 55‐year‐old Ghanaian male with incidentally detected isolated LVNC. This case report highlights the need for a high index of suspicion during echocardiography and the application of appropriate diagnostic criteria in diagnosis‐making. This is particularly important for lower‐middle‐income countries (LMIC), where advanced imaging may be inaccessible.

## 2 Case Report

### 2.1 History and Examination

A 55‐year‐old male patient presented with palpitations for a month at the cardiology clinic. The patient is not hypertensive or diabetic and has no long‐standing illnesses. The palpitations occurred intermittently and lasted a few seconds at a time. There was no associated difficulty in breathing, orthopnea, pedal edema, dizziness, or syncope. The patient had no history of thyroid disease or anxiety disorder and was able to perform all his activities with no limitations. He jogged regularly 4 times per week. His two older siblings were all deceased, as were his parents but the cause of their demise is not known to him. He has two teenage children who are all well and he did not drink alcohol or use illicit drugs.

General physical examination was unremarkable, revealing a normal BMI and normal hemodynamic parameters except for a pulse rate of 40 beats per minute which was regular and of good volume. All other systems revealed no abnormalities on examination.

### 2.2 Investigations and Treatment

As part of his assessment, the basic blood panel, electrolyte levels, liver, kidney, thyroid, lipid panel, and chest X‐ray were requested and found to be normal.

**FIGURE 1:**
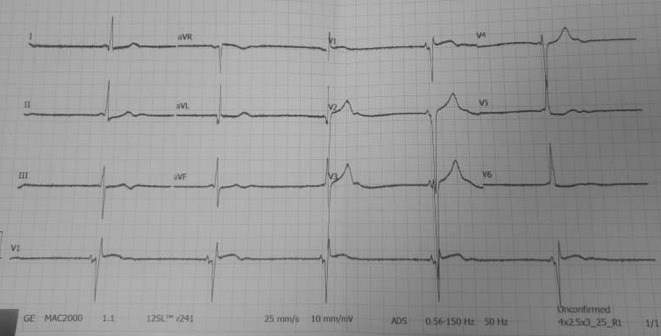
ECG showing sinus bradycardia, short PR interval, and deep QS waves in chest leads V1‐V3.

The electrocardiogram showed sinus bradycardia with a short PR interval and deep QS waves in the anteroseptal leads (Figure 1). 24‐h Holter monitoring showed occasional premature ventricular complexes with couplets against the background of profound sinus bradycardia.

There was a persistent short PR interval with no other conduction abnormalities, atrial or ventricular tachyarrhythmias.

On echocardiography, there was the typical two‐layered appearance of the myocardium with a thin, compacted outer band and a thicker, noncompacted inner layer where prominent LV trabeculae in the mid‐cavity to apical regions with deep recesses communicating with the LV cavity were seen. The ratio of the noncompacted to compacted layers was 2.4 at the apex using the Jenni criterion. No thrombi were seen, the left ventricular ejection fraction was 78% with grade‐one diastolic dysfunction, and no wall motion abnormality to suggest ischemia was noted. All cardiac chambers were of normal dimensions (Video 1).

**VIDEO 1:** Echocardiographic video showing prominent trabeculations in the mid to apical zones of the left ventricle. Video content can be viewed at https://onlinelibrary.wiley.com/doi/10.1002/ccr3.9563

**FIGURE 2:**
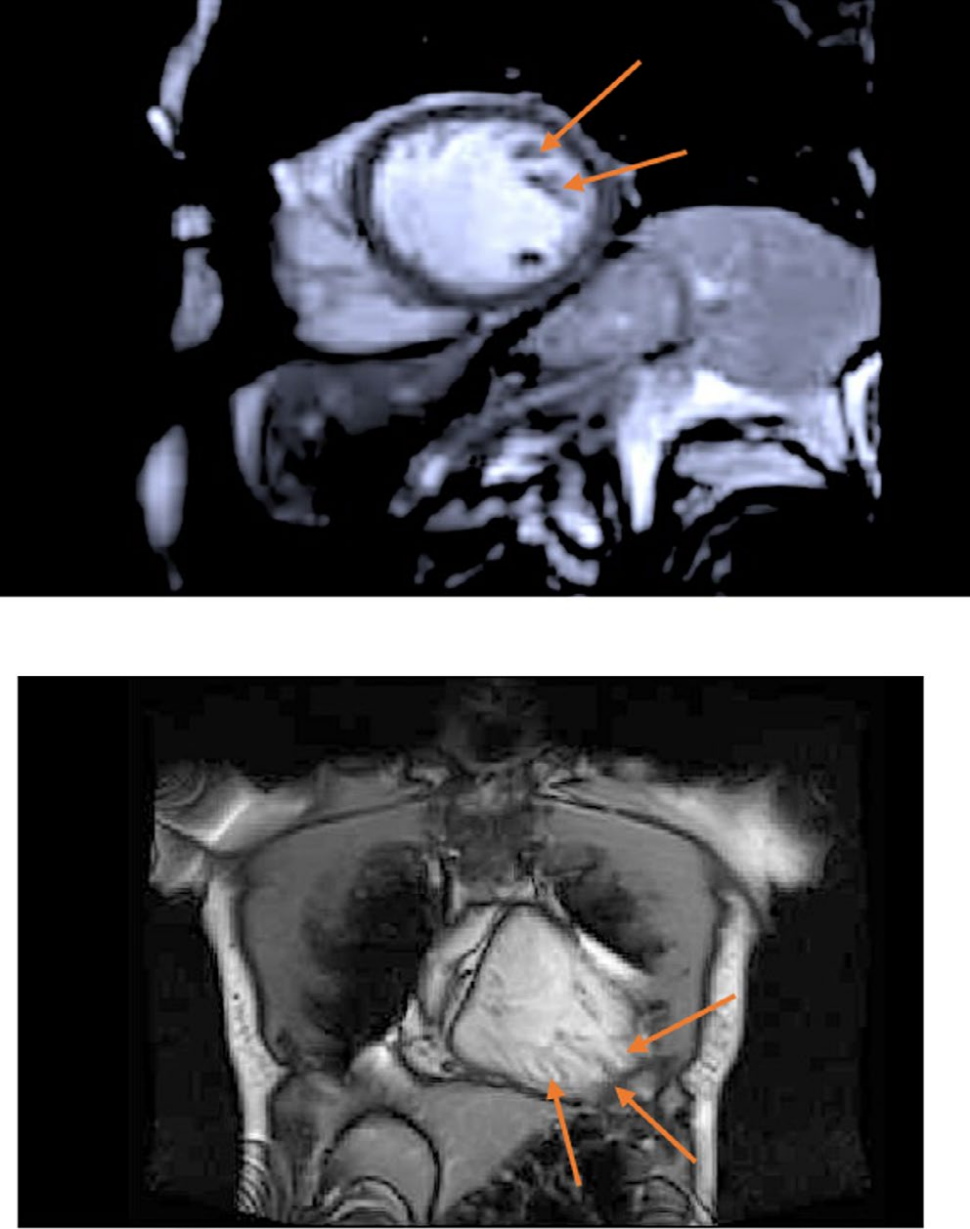
Cardiac MRI images showing trabeculations with deep recesses suggestive of LVNC.

The cardiac magnetic resonance imaging (CMRI) (Figure 2) showed LV good global contractility and mild left ventricular hypertrophy (max 1.2 cm mid‐septum vs. 1.1 cm mid‐lateral wall). There were prominent LV trabeculations with a noncompacted to compacted ratio (NC: C ratio) at the base being 2.3, at the mid cavity, 2.5 and at the apex, 3 consistent with LV noncompaction syndrome using the Petersen criterion. No left ventricular outflow tract (LVOT) obstruction, systolic anterior motion (SAM) phenomenon, or regional wall motion abnormality was seen. No myocardial inflammation was seen, and the cardiac chamber dimensions and function were normal.

2D echocardiography of the two teenage children of the patient was normal.

Subsequently, the patient was counseled on the diagnosis and the likelihood of tachyarrhythmias and bradyarrhythmias. Electrophysiology studies were considered; however, the service is unavailable in Ghana, and the patient could not afford to travel outside the country to do it. With the unavailability of event monitors and loop recorders, the patient and his health care practitioners decided on yearly 48–72‐h Holter recordings to screen for arrhythmias or to do so if palpitations occurred. He is on candesartan 8 mg daily and doing well on three monthly reviews. The palpitations have subsided.

## 3 Conclusion

In this case report, we have described a patient with cardiac MRI‐confirmed LVNC presenting with palpitations. The ECG showed a shortened PR interval and bradycardia that could not be further evaluated on account of unavailability in Ghana. Routine screening of living first‐degree relatives did not indicate LVNC. The patient is assessed yearly with echocardiography and a 48‐h Holter.

The article has explored the use of echocardiography in the early detection of genetic cardiomyopathies, particularly in LMICs, and provided an overview of the assessment of LVNC. In a setting where there is a lack of advanced imaging facilities, it is believed that diligent application of echocardiography, with a high index of suspicion, will enhance the detection of rare genetic abnormalities and initiate their optimal treatment and follow‐up. Asymptomatic relatives of index cases can also be screened, counseled, and followed up to detect complications and offer appropriate, timely. Large, longitudinal studies of African patients with hypertrabeculation and their relatives are needed and will provide invaluable information on prevalence, disease progression, complications, and outcomes and guide the care of such patients. Additionally, the need for other monitoring and advanced imaging expertise and equipment locally has been highlighted by this case report.

## 4 Discussion

Left ventricular noncompaction (LVNC) is a structural disorder of the left ventricle associated with trabeculations in the ventricle and recesses between these trabeculations [9, 10, 11]. Although the American Heart Association classifies LVNC as a genetic cardiomyopathy, the European Society of Cardiology considers it as an unclassified cardiomyopathy [2, 10] with the lack of a standard definition leading to an estimated prevalence of 0.01% to 0.24% [13]. Although LVNC is not uncommon in sub‐Saharan Africa [14, 15], data in Ghana remain scarce.

The exact cause of the LVNC phenotype is unknown. Still, some gene mutations, such as NOTCH genes, ACTC_1_, MYH_7_, MYBPC3, TTN, and HCN4, among others, have been implicated [11, 16] with the HCN4 gene responsible for sinus bradycardia in some patients [16, 18]. Only about 43% of these are familial, which reflects de novo mutations that arise in adults [19]. These defects alter the functions of sarcomeres, Z‐disc, and nuclear envelopes, resulting in impaired cardiac myofibrillogenesis which ultimately halts the compaction of the ventricles during embryogenesis [11, 12].

Screening for LVNC is usually considered in the workup for all first‐degree relatives of individuals with LVNC [20, 21] and can be done through imaging [16, 22] or genetic testing [5, 20]. In South Africa, 83 relatives of 38 unrelated patients with LVNC were screened using ECG and echocardiography. There was a 76% sensitivity and 42% specificity of detection using ECG compared to echocardiography [16].

Although LVNC alters the anatomy of the left ventricular endomyocardium which affects the electrical activity of the heart, LVNC lacks specific features on the ECG making it an unfavorable tool for screening [16]. While a normal ECG is not uncommon, conduction abnormalities like left bundle branch block (LBBB) and AV blocks are the commonest findings [25]. Other reported ECG changes include inverted T waves, Q waves, ventricular ectopics, repolarization abnormalities, QTc prolongation, and ventricular arrhythmias [10]. Electrophysiological studies in LVNC have shown that ventricular arrhythmias originate from the noncompacted layers of the left ventricles and the mid‐apical LV segments, while focal premature ventricular complexes originate from the papillary muscle and the LV basal‐septal regions [26]. Except for sustained monomorphic VTs, most ventricular and supraventricular arrhythmias are also inducible during electrophysiological testing but Steffel et al. did not consider routine testing to be useful [27]. Other potentially useful investigative tools for LVNC include multidetector CT, left ventricular angiography, and endomyocardial biopsies [24, 28].

The 2‐D echocardiogram is useful in low‐middle‐income countries, like Ghana, for the workup of LVNC, due to its relatively low cost and access [14]. It is operator‐dependent, and its usefulness depends on the ability to delineate the apex of the heart [10]. Speckle tracking echocardiography, the use of contrast, strain rate imaging, tissue Doppler images, and 3‐dimensional echocardiography [13, 24] enhance the probability of detection but are unavailable in Ghana.

The Chin criterion was the first to be developed to diagnose LVNC and is based on images obtained from the apical 4 chamber or parasternal long axis, during the end‐diastolic phase of the cardiac cycle [10]. A diagnostic ratio of ≤ 0.5, calculated as the distance from the deepest recess to the epicardial surface, to the distance from the tip of a trabeculation to the epicardial surface, has a sensitivity and specificity of 79%–100% and 54%–92%, respectively [10]. The Stōllberger criterion relies on images obtained in the apical 4‐chamber view, during end‐diastole. It is considered diagnostic when 3 or more trabeculations with synchronous movement during systole and similar echogenicity to the myocardium are seen from the papillary muscles in the left ventricle [10]. To be valid, a ratio of the compacted layer to the noncompacted layer is expected to be > 2, with blood flow seen between trabeculations [29]. The Jenni criteria is considered diagnostic when blood is seen between trabeculation. At the same time, other cardiac abnormalities are ruled out, and the ratio of the noncompacted layer to the compacted layer is > 2 [29]. Unlike the previously described criteria, the Jenni criteria are obtained from the parasternal short axis and during the end‐systolic phase of the cardiac cycle [29].

In a study by Joong et al., which compared the utility and reproducibility among three criteria, the Jenni, Stollberger, and Chin criteria, it was found that the parasternal short‐axis echocardiographic view offered the most reliability, especially with an assessment of apical trabeculae. They also noted that despite the widespread use of the Jenni criterion, the Chin criterion has the highest sensitivity of 79%–100% and the highest specificity of 54%–92% [34].

Similar studies are required among African patients with LVNC to determine the echocardiographic criterion with the highest specificity and sensitivity to enhance echocardiographic diagnostic accuracy in LMICs.

The CMR provides added information such as the right ventricle function, better images of the noncompacted myocardium during diastole, and the presence of fibrosis [3, 24]. Demerits include a relatively longer time to obtain images, the need for the patient to remain supine during the procedure, and cost‐driven inaccessibility in LMIC [14].

In a study that compared 4 CMR‐based criteria for the diagnosis of LVNC (Stacy, Petersen, Jacquier, and Captur), the prevalence was found to be 23%, 39%, 25%, and 3%, respectively [35], indicating that even using the gold standard imaging technique, there are significant variations in detection rates.

The Petersen criteria, being the commonest, are considered diagnostic when the ratio of the noncompacted layers to the compacted, in the end‐diastolic phase, is > 2.3 [10]. The noncompacted layers are measured perpendicular to the compacted layers and are better delineated in end‐diastole [29]. In 2020, a study comparing echocardiography (Jenni criterion) versus CMR (Petersen and Jacquier criteria) in the assessment of LVNC indicated that the best cut‐off for making the diagnosis was noncompacted to compacted ratio at or above 2.3 in the Petersen criterion and a trabeculated LV mass at or above 20% with the Jacquier criterion. On 2D echocardiography, the Jenni criterion was applied with the noncompacted to compacted ratio of ≥ 1.8 (instead of 2.0). This echocardiographic criterion was associated with a sensitivity of 98% and a specificity of 100% [17]. The decision to apply the Jenni criterion to our patient was based on this study.

The prognosis of LVNC is variable and is based on the clinical presentations [3]. It ranges from asymptomatic states [5] commonly seen in adults to worse outcomes in children, partly due to concomitant congenital and developmental abnormalities [19]. Management of LVNC is largely based on symptoms as there are currently no known effective treatment strategies [5]. Heart failure is managed with the use of guideline‐directed medical therapy, beta‐blockers alone being associated with improved quality of life in adult patients [5]. Left ventricular assist device, cardiac resynchronization, and heart transplant can also be offered when indicated [3].

Ventricular tachyarrhythmias are the most common arrhythmias in LVNC, followed by atrial fibrillation [30] and all these arrhythmias are managed as per their guidelines. Implantable cardioverter defibrillator and radiofrequency catheter ablation can be considered if patients are symptomatic despite optimal medical therapy [29]. The use of ICDs for primary prevention of SCD remains controversial, and its use is recommended as per general guidelines for the use of ICDs even though patients with an increased risk for SCD can be considered [10]. LVNC patients are also at risk of thromboembolic events, even in the absence of atrial fibrillation but the routine use of anticoagulants for primary prevention remains controversial [3, 30]. Other causes of thromboembolism in LVNC include systolic dysfunction, as well as trabeculations [31]. Accepted indications for primary prevention include atrial fibrillation, as per the CHADS2VASc score, and patients with reduced LVEF [32].

Mortality from LVNC is variable and considered to be similar to that of nonischemic dilated cardiomyopathy [33]. Sudden cardiac death and heart failure are the commonest causes of death among LVNC patients, while thromboembolism and arrhythmias contribute to their morbidities [14].

## Author Contributions

**Aba A. Folson:** conceptualization, supervision, writing – original draft, writing – review and editing. **Philip Eghan:** conceptualization, writing – review and editing. **Daniel Amenu:** conceptualization, writing – review and editing.

## Consent

Written consent has been obtained from the patient described in this case report and will be made available to the publishers upon request.

## Supporting information


Data S1.

Table S1.

## Data Availability

The data that support the findings of this study are available on request from the corresponding author. The data are not publicly available due to privacy or ethical restrictions.

## References

[1] F. Fusco , N. Borrelli , R. Barracano , et al., “Left Ventricular Non‐Compaction Spectrum in Adults and Children: From a Morphological Trait to a Structural Muscular Disease,” Cardiogenetics 12 (2022): 170–184, https://www.mdpi.com/2035‐8148/12/2/16/htm.

[2] E. Arbelo , A. Protonotarios , J. R. Gimeno , et al., “ESC Guidelines for the Management of Cardiomyopathies: Developed by the Task Force on the Management of Cardiomyopathies of the European Society of Cardiology (ESC),” European Heart Journal ‐ Cardiovascular Pharmacotherapy 44, no. 37 (2023): 3503–3626, 10.1093/eurheartj/ehad194. 37622657

[3] J. A. Towbin and J. L. Jefferies , “Cardiomyopathies Due to Left Ventricular Noncompaction, Mitochondrial and Storage Diseases, and Inborn Errors of Metabolism,” Circulation Research 121, no. 7 (2017): 838–854, 10.1161/CIRCRESAHA.117.310987. 28912186

[4] N. D. Gaye , A. A. Ngaïdé , M. B. Bah , K. Babaka , A. Mbaye , and K. Abdoul , “Non‐compaction of Left Ventricular Myocardium in Sub‐Saharan African Adults,” Heart Asia 9, no. 2 (2017): e010884, 10.1136/heartasia-2017-010884. 29467831 PMC5818043

[5] D. Li and C. Wang , “Advances in Symptomatic Therapy for Left Ventricular Non‐compaction in Children,” Frontiers in Pediatrics 11 (2023): 1147362. 37215603 10.3389/fped.2023.1147362 PMC10192632

[6] G. Femia , C. Semsarian , S. B. Ross , D. Celermajer , and R. Puranik , “Left Ventricular Non‐Compaction: Review of the Current Diagnostic Challenges and Consequences in Athletes,” Medicina (Kaunas, Lithuania) 56, no. 12 (2020): 1–10. 10.3390/medicina56120697 PMC7764920 33327510

[7] F. Peters and M. R. Essop , “Isolated Left Ventricular Non‐compaction in Africa: Elucidating Myths,” Cardiovascular Journal of Africa 24, no. 5 (2013): 188. 24217167 PMC3749492

[8] F. Zuccarino , I. Vollmer , G. Sanchez , M. Navallas , F. Pugliese , and A. Gayete , “Left Ventricular Noncompaction: Imaging Findings and Diagnostic Criteria,” AJR. American Journal of Roentgenology 204, no. 5 (2015): W519–W530, 10.2214/AJR.13.12326. 25905958

[9] K. Hirono and F. Ichida , “Left Ventricular Noncompaction: A Disorder With Genotypic and Phenotypic Heterogeneity—A Narrative Review,” Cardiovascular Diagnosis &Ttherapy 12, no. 4 (2022): 495–515, https://cdt.amegroups.org/article/view/98990/html. 10.21037/cdt-22-198 PMC9412206 36033229

[10] J. Paluszkiewicz , H. Milting , M. Kałużna‐Oleksy , et al., “Left Ventricular Non‐Compaction Cardiomyopathy‐Still More Questions Than Answers,” Journal of Clinical Medicine 11, no. 14 (2022): 4135, https://www.mdpi.com/2077‐0383/11/14/4135/htm. 35887898 10.3390/jcm11144135 PMC9315982

[11] P. Rojanasopondist , L. Nesheiwat , S. Piombo , G. A. Porter , M. Ren , and C. K. L. Phoon , “Genetic Basis of Left Ventricular Noncompaction,” Circulation: Genomic and Precision Medicine 15, no. 3 (2022): E003517, 10.1161/CIRCGEN.121.003517. 35549379

[12] S. Nel , B. K. Khandheria , E. Libhaber , et al., “Prevalence and Significance of Isolated Left Ventricular Non‐compaction Phenotype in Normal Black Africans Using Echocardiography,” International Journal of Cardiology. Heart & Vasculature 30 (2020): 100585, 10.1016/j.ijcha.2020.100585. 32715082 PMC7378683

[13] S. Gati , R. Rajani , G. S. Carr‐White , and J. B. Chambers , “Adult Left Ventricular Noncompaction: Reappraisal of Current Diagnostic Imaging Modalities,” JACC: Cardiovascular Imaging 7, no. 12 (2014): 1266–1275. 25496545 10.1016/j.jcmg.2014.09.005

[14] O. S. Ogah , E. P. Iyawe , O. A. Orimolade , et al., “Left Ventricular Noncompaction in Ibadan, Nigeria,” Egyptian Heart Journal 75, no. 1 (2023): 69, 10.1186/s43044-023-00396-9. PMC10415240 37563298

[15] F. Peters , B. K. Khandheria , C. Dos Santos , et al., “Isolated Left Ventricular Noncompaction in Sub‐Saharan Africa: A Clinical and Echocardiographic Perspective,” Circulation. Cardiovascular Imaging 5, no. 2 (2012): 187–193, 10.1161/CIRCIMAGING.111.966937. 22235038

[16] A. L. Basson , M. R. Essop , E. Libhaber , and F. Peters , “Family Screening in Black Patients With Isolated Left Ventricular Non‐compaction: The Chris Hani Baragwanath Experience,” Cardiovascular Journal of Africa 31, no. 4 (2020): 180–184, 10.5830/CVJA-2020-003. 32159583 PMC8762831

[17] V. Donghi , F. Tradi , A. Carbone , et al., “Left‐Ventricular Non‐compaction‐Comparison Between Different Techniques of Quantification of Trabeculations: Should the Diagnostic Thresholds Be Modified?,” Archives of Cardiovascular Diseases 113, no. 5 (2020): 321–331, 10.1016/j.acvd.2020.01.004. 32249166

[18] A. Paszkowska , D. Piekutowska‐Abramczuk , E. Ciara , et al., “Clinical Presentation of Left Ventricular Noncompaction Cardiomyopathy and Bradycardia in Three Families Carrying HCN4 Pathogenic Variants,” Genes (Basel) 13, no. 3 (2022): 477. 35328031 10.3390/genes13030477 PMC8949387

[19] J. I. van Waning , J. Moesker , D. Heijsman , E. Boersma , and D. Majoor‐Krakauer , “Systematic Review of Genotype‐Phenotype Correlations in Noncompaction Cardiomyopathy,” Journal of the American Heart Association 8, no. 23 (2019): e012993, 10.1161/JAHA.119.012993. 31771441 PMC6912966

[20] Y. M. Hoedemaekers , K. Caliskan , M. Michels , et al., “The Importance of Genetic Counseling, DNA Diagnostics, and Cardiologic Family Screening in Left Ventricular Noncompaction Cardiomyopathy,” Circulation. Cardiovascular Genetics 3, no. 3 (2010): 232–239, 10.1161/CIRCGENETICS.109.903898. 20530761

[21] G. Casas , G. Oristrell , J. Rodriguez‐Palomares , et al., “P2250Role of Family Screening and Genetic Testing in Left Ventricular Noncompaction,” European Heart Journal 39, no. suppl_1 (2018): ehy565‐P2250, 10.1093/eurheartj/ehy565.P2250.

[22] J. G. Dreisbach , S. Mathur , C. P. Houbois , et al., “Cardiovascular Magnetic Resonance Based Diagnosis of Left Ventricular Non‐compaction Cardiomyopathy: Impact of Cine bSSFP Strain Analysis,” Journal of Cardiovascular Magnetic Resonance 22, no. 1 (2020): 1–14, 10.1186/s12968-020-0599-3. 31996239 PMC6990516

[23] J. L. Jefferies , “Left Ventricular Noncompaction Cardiomyopathy: New Clues in a Not So New Disease?,” Journal of the American Heart Association 10, no. 2 (2021): 1–3, 10.1161/JAHA.120.018815. PMC7955298 33442993

[24] C. Bustea , A. F. Bungau , D. M. Tit , et al., “The Rare Condition of Left Ventricular Non‐Compaction and Reverse Remodeling,” Life 13, no. 6 (2023): 1318. 37374101 10.3390/life13061318 PMC10305066

[25] E. Purevjav , M. Chintanaphol , B.‐O. Orgil , et al., “Left Ventricular Noncompaction Cardiomyopathy: From Clinical Features to Animal Modeling,” in Preclinical Animal Modeling in Medicine (London: IntechOpen, 2021), https://www.intechopen.com/chapters/79211.

[26] D. Muser , J. J. Liang , W. R. Witschey , et al., “Ventricular Arrhythmias Associated With Left Ventricular Noncompaction: Electrophysiologic Characteristics, Mapping, and Ablation,” Heart Rhythm 14, no. 2 (2017): 166–175. 27890738 10.1016/j.hrthm.2016.11.014

[27] J. Steffel , R. Kobza , E. Oechslin , R. Jenni , and F. Duru , “Electrocardiographic Characteristics at Initial Diagnosis in Patients With Isolated Left Ventricular Noncompaction,” American Journal of Cardiology 104, no. 7 (2009): 984–989, http://www.ajconline.org/article/S0002914909011229/fulltext. 19766768 10.1016/j.amjcard.2009.05.042

[28] B. J. Gerecke and R. Engberding , “Noncompaction Cardiomyopathy—History and Current Knowledge for Clinical Practice,” Journal of Clinical Medicine 10, no. 11 (2021): 2457. 34206037 10.3390/jcm10112457 PMC8199228

[29] U. Ikeda , M. Minamisawa , and J. Koyama , “Isolated Left Ventricular Non‐compaction Cardiomyopathy in Adults,” Journal of Cardiology 65, no. 2 (2015): 91–97. 25468766 10.1016/j.jjcc.2014.10.005

[30] F. Sedaghat‐Hamedani , J. Haas , F. Zhu , et al., “Clinical Genetics and Outcome of Left Ventricular Non‐compaction Cardiomyopathy,” European Heart Journal 38, no. 46 (2017): 3449–3460, 10.1093/eurheartj/ehx545. 29029073

[31] C. Cevik , N. Shah , J. M. Wilson , and R. F. Stainback , “Multiple Left Ventricular Thrombi in a Patient With Left Ventricular Noncompaction,” Texas Heart Institute Journal 39, no. 4 (2012): 550. 22949776 PMC3423290

[32] C. E. Bennett and R. Freudenberger , “The Current Approach to Diagnosis and Management of Left Ventricular Noncompaction Cardiomyopathy: Review of the Literature,” Cardiology Research and Practice 2016 (2016): 5172308. 26881173 10.1155/2016/5172308 PMC4737020

[33] N. Aung , S. Doimo , F. Ricci , et al., “Prognostic Significance of Left Ventricular Noncompaction,” Circulation. Cardiovascular Imaging 13 (2020): e009712, 10.1161/CIRCIMAGING.119.009712. 31959004 PMC7012350

[34] S. Srivastava , M. Yavari , A. Al‐Abcha , S. Banga , and G. Abela , “Ventricular Non‐compaction Review,” Heart Failure Reviews 27, no. 4 (2022): 1063–1076, 10.1007/s10741-021-10128-3. 34232438

[35] A. Ivanov , D. S. Dabiesingh , G. P. Bhumireddy , et al., “Prevalence and Prognostic Signifcance of Left Ventricular Noncompaction in Patients Referred for Cardiac Magnetic Resonance Imaging,” Circulation. Cardiovascular Imaging 10, no. 9 (2017): e006174. 28899950 10.1161/CIRCIMAGING.117.006174

